# Supplementary material for: Tead1a Initiates Transcriptional Priming Through the TEAD1a/YAP‐Notch1‐Spi1/Cebpα Axis to Promote Neutrophil Fate
Source: Adv Sci (Weinh). 2025 Aug 28;12(41):e05441. doi: 10.1002/advs.202505441 (PMC12591127; doi:10.1002/advs.202505441)
Supplement: Supplementary file 4 — Supplementary Dataset [file ADVS-12-e05441-s004.zip › 3-3.zip!/3-3/02-3.cluster/cluster7/Enrich/ego_MF.pdf]

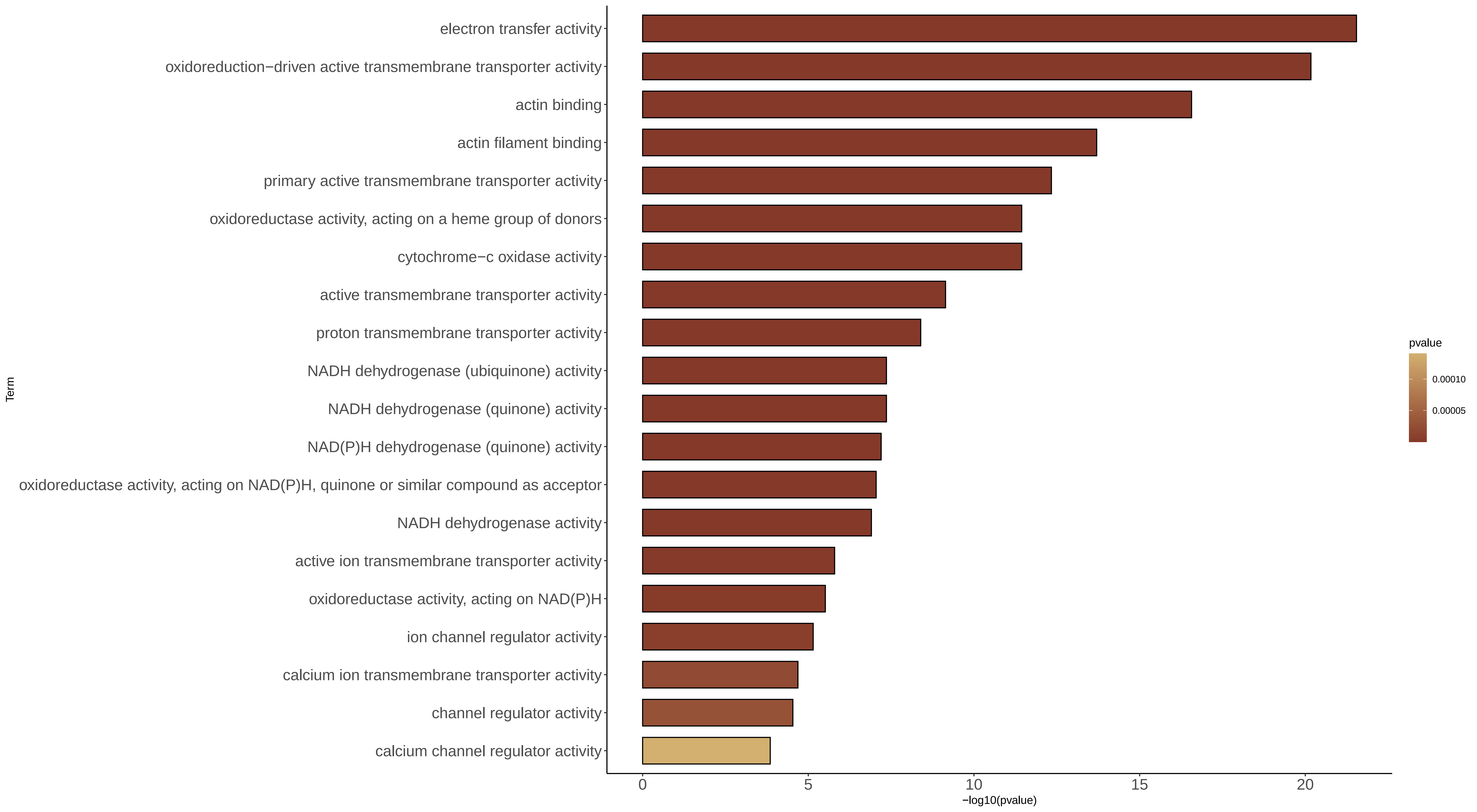

electron transfer activity
oxidoreduction−driven active transmembrane transporter activity
actin binding
actin filament binding
primary active transmembrane transporter activity
oxidoreductase activity, acting on a heme group of donors
cytochrome−c oxidase activity
active transmembrane transporter activity
proton transmembrane transporter activity
NADH dehydrogenase (ubiquinone) activity
NADH dehydrogenase (quinone) activity
NAD(P)H dehydrogenase (quinone) activity
oxidoreductase activity, acting on NAD(P)H, quinone or similar compound as acceptor
NADH dehydrogenase activity
active ion transmembrane transporter activity
oxidoreductase activity, acting on NAD(P)H
ion channel regulator activity
calcium ion transmembrane transporter activity
channel regulator activity
calcium channel regulator activity
Term
0
5
10
15
20
−log10(pvalue)
pvalue
0.00010
0.00005
